# *Enterococcus gallinarum* meningitis: a case report and literature review

**DOI:** 10.1186/s12879-018-3151-4

**Published:** 2018-05-21

**Authors:** Bo Zhao, Mao Sheng Ye, Rui Zheng

**Affiliations:** Department of Respiratory Medicine, Shengjing Hospital of China Medical University, Shenyang, China

**Keywords:** *Enterococcus gallinarum* meningitis, Infections

## Abstract

**Background:**

As an opportunistic pathogen, *E. gallinarum* mainly leads to nosocomial infections, and it’s multi-drug resistance has gained more and more attention. Central nervous system infections caused by *E. gallinarum* are rare, but have been reported more often in recent years. The previous cases were generally secondary to neurosurgery, especially ventriculoperitoneal shunts. In recent years, the cases largely occurred in patients with impaired immune function. The patient in our report may have had dual risk factors (immune impairment and an invasive surgical procedure).

**Case presentation:**

The patient, a 35-year-old female, was admitted to our hospital for headaches of 3 days duration accompanied by nausea and vomiting for 2 days. The patient had fevers and chills for 3 days before admission; the peak body temperature was 38.5 °C. The patient had a splenectomy in our hospital 2 years earlier for thrombocytopenia and was thought to be immunocompromised. The abnormal findings on physical examination and laboratory testing were as follows: neck stiffness, present; lumbar puncture: pressure, 300 mmH_2_O; Pandy’s test, positive; white blood cell (WBC) count, 1536 × 10^6^/L; monocyte count, 602 × 10^6^/L; monocyte percentage, 39.2%; multinucleate cell count, 934 × 10^6^/L; multinucleate cell percentage, 60.8%; protein, 1.08 g/L; WBC count, 21.1 × 10^9^/ L; neutrophil percentage, 85.3%; neutrophil count, 20.55 × 10^9^/L; C reactive protein (CRP): 136.4 mg/L; procalcitonin, 6.70 ng/mL. The patient was given meropenem (2.0 g, intravenous infusion, every 8 h) for anti-infection supplemented with other symptomatic support treatments. The patient’s fever and headache had no significant relief.

**Conclusions:**

Central nervous system infections caused by E. gallinarum are rare, but should be suspected, particularly inpatients with impaired immune function or ineffective treatment. Avoiding long-term invasive treatment and improving immunity are helpful to reduce the occurrence of *E. gallinarum* infections. Early detection and diagnosis, as well as rational antibiotic use, are the keys to achieve satisfactory efficacy.

## Background

As an opportunistic pathogen, *E. gallinarum* mainly leads to nosocomial infections, and it’s multi-drug resistance has gained more and more attention. Central nervous system infections caused by *E. gallinarum* are rare, but have been reported more often in recent years. The previous cases were generally secondary to neurosurgery, especially ventriculoperitoneal shunts. In recent years, the cases largely occurred in patients with impaired immune function. The patient in our report may have had dual risk factors (immune impairment and an invasive surgical procedure).

## Case presentation

The patient, a 35-year-old female, was admitted to our hospital for evaluation of headaches of 3 days duration accompanied by nausea and vomiting for 2 days. The patient had fevers and chills for 3 days before admission; the peak body temperature was 38.5 °C. 2 days before admission, the patient developed headaches, which were persistent and intolerable, accompanied by four episodes of vomiting. The patient had a splenectomy in our hospital 2 years earlier for thrombocytopenia and was thought to be immunocompromised. The findings on physical examination, imaging, and laboratory testing after admission were as follows: skin and mucous, normal; heart, lung, and abdomen, normal; neck stiffness, present; Kernig’s sign, negative; lumbar puncture: pressure, 300 mmH_2_O; Pandy’s test, positive; white blood cell (WBC) count, 1536 × 10^6^/L; monocyte count, 602 × 10^6^/L; monocyte percentage, 39.2%; multinucleate cell count, 934 × 10^6^/L; multinucleate cell percentage, 60.8%; protein, 1.08 g/L (Table 1); head and chest CT, normal; head contrast MRI + MRA + MRV, normal; WBC count, 21.1 × 10^9^/ L; neutrophil percentage, 85.3%; neutrophil count, 20.55 × 10^9^/L; C reactive protein (CRP): 136.4 mg/L; procalcitonin, 6.70 ng/mL; liver and kidney function, normal; and electrolytes, normalMeropenem (2.0 g intravenous infusion every 8 h) was administered with other symptomatic support treatments, such as reducing intracranial pressure by mannitol. The temperature fluctuated around 38 °C. There was no significant relief from the headaches. A lumbar puncture was repeated 6 days after admission. The cerebrospinal fluid culture and drug sensitivity testing showed an *Enterococcus gallinarum* infection and sensitivity to linezolid (Table 2), respectively. Thus, an intravenous infusion of linezolid (0.6 g every 12 h) was administered. On the second day of linezolid, the temperature began to decrease. After 3 weeks of anti-*E. gallinarum* treatment, the temperature returned to normal and the headache resolved. A lumbar puncture was repeated three times. The cerebrospinal fluid was colorless and transparent, the pressure and WBC count were decreased, and the bacterial cultures were negative. The patient was discharged from the hospital when stable and in good condition.

**Table 1:** Results of lumbar puncture after admission

Lumbar puncture	1st day	6th day	14th day	22nd day	31st day
Pressure mmH_2_O (80–180)	300	300	160	110	110
Appearance (Colorless and transparent)	Colorless and transparent	Light yellow and transparent	Colorless and transparent	Colorless and transparent	Colorless and transparent
Pandy’s test (−)	+	+	Weak positive	Weak positive	–
WBC count 10^6^/L (0–8)	1536	204	107	36	11
Monocyte count 10^6^/L (not available)	602	164	106	36	10
Monocyte percentage % (not available)	39.2	92.1	99.1	100.0	97.9
Multinucleate cell count 10^6^/L (not available)	934	40	1	0	1
Multinucleate cell percentage % (not available)	60.8	7.9	0.9	0	2.1
RBC count 10^6^/L (0)	0	0	0	0	0
Glucose mmol/L (2.5–4.5)	3.21 (RBG 6.80)	2.32 (BG not tested)	3.33 (FBG 5.71)	3.03 (BG not tested)	3.1 (FBG 4.54)
Chlorine mmol/L (120–132)	121.5	115	120.0	118.2	119.7
Protein g/L (0.15–0.45)	1.08	0.84	0.52	0.41	0.33
*Cryptococcus* smear (Ink stain)	–	–	–	–	–
*Mycobacterium tuberculosis* smear (Acid-fast stain)	–	–	–	–	–
Bacterial smear (Gram’s stain)	–	–	–	–	–
Bacterial culture (Plate cultivation)	–	*Enterococcus gallinarum*	–	–	–

*BG* blood glucose, *RBG* random blood glucose, *FBG* fasting blood glucose

**Table 2:** The susceptibility results of *E.gallinarum*

Antibiotic name	Method	Result	Sensitivity	Determination standard		
				Sensitive	Intermediary	Resistance
Penicillin G	MIC	8.0	S	≥16		≤8
Vancomycin	MIC	2.0	R	≥32	8–16	≤4
Linezolid	MIC	1.0	S	≥8	4	≤2
Tetracycline	MIC	≥16.0	R	≥16	8	≤4
Ciprofloxacin	MIC	≤0.5	S	≥4	2	≤1
Erythromycin	MIC	8.0	R	≥8	1–4	≤0.5
Levofloxacin	MIC	1.0	S	≥8	4	≤2
Ampicllin	MIC	≤2.0	S	≥16		≤8
Quinupristin/Dalfopristin	MIC	1.0	R	≥4	2	≤1
Clindamycin	MIC	≥8.0	R	≥4	1–2	≤0.5
Moxifloxacin	MIC	≤0.25	S	≥4	2	≤1
Tigecycline	MIC	≤0.12	S			≤0.25
Gentamicin-High	MIC		S			
Streptomycin-High	MIC		R			

*MIC* minimal inhibitory concentration, *R* resistance, *S* sensitive

## Discussion and conclusions

*Enterococcus gallinarum* was first isolated from the gut of a chicken. *Enterococcus gallinarum* is normal flora in human and animal guts [1]. In recent years, with the increasing use of broad-spectrum antibiotics and invasive medical devices, infections caused by *E. gallinarum* have gradually increased, and multi-drug resistance has gained more and more attention. In 2010, among the isolated strains of *Enterococcus* in several Chinese hospitals, *E. gallinarum* accounted for 1.9% of isolates, and second only to *E. faecalis* and *E. faecium* [2]. As an opportunistic pathogen, *E. gallinarum* mainly leads to nosocomial infections, including urinary tract, abdominal, biliary tract, and a small percentage of bloodstream infections. Patients who undergo invasive operations or are immunosuppressed are susceptible [3, 4]. Central nervous system infections caused by *E. gallinarum* are rare, but have been reported more often in recent years.

Symptoms of *E. meningitis* include fevers and headaches, which may be accompanied by a disturbance of consciousness or even convulsions. Some patients may have septic shock, focal neurologic deficits, petechial rashes, and meningeal irritation [4]. High value of CRP and procalcitonin can be found in patients with *E. gallinarum* meningitis. The diagnosis of *E. gallinarum* meningitis is based on clinical symptoms, cerebrospinal fluid examination, and pathogen culture. PCR is also used for diagnosis, the results of which can be obtained 48 h earlier than routine bacterial cultures [5]. The patient in this report exhibited fevers, headaches, and neck stiffness. The cerebrospinal fluid was purulent and the culture confirmed an infection with *E. gallinarum*. The patient had undergone a splenectomy and her immunoglobulin level was lower than the normal value, suggesting impairment of humoral immune function, which increased her risk for opportunistic infections [6]. The cerebrospinal fluid culture after the first lumbar puncture was negative, and the possibility that the pathogen was introduced by the first lumbar puncture could not be excluded. Moreover, the administration of broad-spectrum antibiotics may have exacerbated the infection.

There have been eight *E. gallinarum* meningitis cases reported worldwide (Table 3). The previous cases were generally secondary to neurosurgery, especially ventriculoperitoneal shunts. In recent years, the cases largely occurred in patients with impaired immune function. The patient in our report may have had dual risk factors (immune impairment and an invasive surgical procedure).

**Table 3:** *Enterococcus gallinarum* meningitis reports in the literature

Reference	Country	Gender	Age	Symptoms	Susceptibility factors	Treatment	Outcome
Yoko Takayama, et al. [8] 2003	Japan	Male	57 years	Fever Neck stiffness	VP shunt for subarachnoid hemorrhage Rheumatoid arthritis with prednisolone and anti-rheumatic drugs	i.v. teicoplanin for 4 weeks VP shunt removal	Cured
Yoko Takayama. et al. [8] 2003	Japan	Male	12 years	Fever Drowsy Limb cramps	VP shunt for astrocytoma	i.v. ampicillin for 8 weeks VP shunt replaced	Cured
Asok Kurup, et al. [9] 2001	Singapore	Male	64 years	Fever Drowsy	VP shunt for multi-loculated hydrocephalus	i.v. ampicillin and gentamicin for 3 weeks	Cured
Fahmi Yousef Khan, et al. [10] 2011	Pakistan	Female	53 years	Fever Headache Consciousness disturbance Neck stiffness	Decompression craniotomy for cerebral hemorrhage	i.v. linezolid for 3 weeks	Cured
Vicente Sperb Antonello, et al. [11] 2010	Brazil	Male	53 years	Mental confusion Fever Ataxia Neck stiffness	Alcohol abuse	i.v. ampicillin and gentamycin for 3 weeks	Cured
B. Roca, et al. [12] 2006	Spain	Female	51 years	Fever Headache	Cerebrospinal fluid drainage catheter for persistent right nostril rhinorrhea	i.v. ampicillin and rifampin for 3 weeks Drain removal	Cured
Po-Yi Paul Su, et al. [5] 2016	USA	Male	53 years	Fever Neck stiffness	Acute lymphoblastic B cell leukemia with chemotherapy Neutropenic Broad-spectrum antibiotics usage Type 2 diabetes mellitus	i.v. ampicillin and ceftriaxone for 4 weeks	Cured
Quanxiao Li, et al. [13] 2013	China	Male	2 days	Fever Hypermyotonia	Neonatal hemolysis	i.v. linezolid for 3 weeks	Cured

*VP shunt* ventriculoperitoneal shunt, *i.v* intravenous

*Enterococcus gallinarum* carries the vanC drug-resistance gene and has a high rate of resistance for vancomycin (82.1%). The pathogen is relatively sensitive to teicoplanin and linezolid [2]. The strains carrying the vanA or vanB resistance genes have been isolated, and are resistant to vancomycin and teicoplanin.[7]. Based on drug sensitivity testing, we chose linezolid at an adequate dose and time to treat the patient. The course of linezolid generally lasts 3 weeks or longer, and the prognosis is good. We recommended a 3-week course of linezolid and obtained satisfactory efficacy. The symptoms, signs, and follow-up results of the cerebrospinal fluid were all remarkably improved after treatment. The patient did not relapse after treatment was completed.

Avoiding long-term invasive treatment and improving immunity are helpful to reduce the occurrence of *E. gallinarum* infections. Early detection and diagnosis, as well as rational antibiotic use, are the keys to achieve satisfactory efficacy.

## Abbreviations

BG: Blood glucose
CRP: C reactive protein
CT: Computed tomography
FBG: Fasting blood glucose
i.v.: Intravenous
MIC: Minimal inhibitory concentration
MRA: Magnetic Resonance Angiography
MRI: Magnetic Resonance Imaging
MRV: Magnetic Resonance Venography
R: Resistance
RBC: Red Blood Cell
RBG: Random blood glucose
S: Sensitive
VP shunt: Ventriculoperitoneal shunt
WBC: White blood cell
